# Research on RSS Data Optimization and DFL Localization for Non-Empty Environments

**DOI:** 10.3390/s18124419

**Published:** 2018-12-13

**Authors:** Wenyu Mao, Rongxuan Shen, Ke Wang, Guoliang Gong, Yi Xiao, Huaxiang Lu

**Affiliations:** 1 Lab of Artificial Networks, Institute of Semiconductors, CAS, Beijing 100083, China; rxshen@semi.ac.cn (R.S.); wangke2013@semi.ac.cn (K.W.); gongmianjie@semi.ac.cn (G.G.); xiaoyi@semi.ac.cn (Y.X.); luhx@semi.ac.cn (H.L.); 2 CAS Center for Excellence in Brain Science and Intelligence Technology, Shanghai 200031, China; 3 University of Chinese Academy of Sciences, Beijing 100049, China; 4 Beijing Key Laboratory of Semiconductor Neural Network Intelligent Sensing and Computing Technology, Beijing 100083, China

**Keywords:** device-free localization (DFL), RSS filtering, data correlation, ensemble learning

## Abstract

Device-free localization (DFL) is a new technique which can estimate the target location through analyzing the shadowing effect on surrounding radio frequency (RF) links. In a relatively complex environment, the influences of random disturbance and the multipath effect are more serious. There are kinds of noises and disturbances in the received signal strength (RSS) data of RF links and the data itself can even be distorted, which will seriously affect the DFL accuracy. Most of the common filtering methods adopted in DFL field are not targeted and the filtering effects are unstable. This paper researches the characteristics of RSS data with random disturbances and proposes two-dimensional double correlation (TDDC) distributed wavelet filtering. It can filter out the random disturbances and noise while preserving the RSS fluctuations which are helpful for the DFL, thus improving the quality of RSS data and localization accuracy. Furthermore, RSS variation rules for the links are different in complex environments and hence, it is difficult for the collected training samples to cover all possible patterns. Therefore, a single machine learning model with poor generalization ability finds it difficult to achieve ideal localization results. In this paper, the Adaboost.M2 ensemble learning model based on the Gini decision tree (GDTE) is proposed to improve the generalization ability for unknown patterns. Extensive experiments performed in two different drawing rooms demonstrate that the TDDC distributed wavelet filtering and the GDTE localization model have obvious advantages compared with other methods. The localization accuracy rates of 87% and 95% can be achieved respectively in the two environments.

## 1. Introduction

Device-free Localization (DFL) based on the shadow effect of the received signal strength (RSS) has been widely studied [1]. The localization result of DFL is greatly influenced by the layout of the monitoring area. Generally, a household environment—which has various building structures, materials, and sizes of furniture—can be regarded as a complex indoor environment and is different from specific experimental environments. Hence, the deep fade and anti-fade of RF links [2], and the multipath effect of the RF signal in different indoor environments will be very different. 

All kinds of noises and distortions will be mixed into the collected RSS data. It is difficult to predict the time-frequency characteristics of disturbances and noises due to their variety. Their occurrences are random, and the frequencies and amplitudes are varied and unknown. The fluctuations of the RSS data caused by the above disturbances have a great influence on the localization accuracy, and the characteristics of these events are difficult to estimate. We call such fluctuations random disturbances or abnormal fluctuations in this paper.

In the localization process, the moving speed of the personnel target is not constant, and the swings of different parts of the body are various. These also make RSS data fluctuate. And the time-frequency characteristics of these fluctuations are difficult to estimate too. However, unlike the above-mentioned fluctuations, these are helpful and meaningful to localization, hence, we call them normal or helpful fluctuations in this paper. 

Overall, the frequencies and amplitudes of normal fluctuations and random disturbances are unknown and difficult to distinguish through common filtering methods.

At present, many researchers have focused on the localization method and model, but there have been few studies on filtering and optimization for RSS data specifically. The simple or common filters such as moving average, Gaussian filtering, wavelet filtering, etc. [2,3] do not take into account the specific relationship between the two kinds of fluctuations mentioned above, so their effects are unstable. In relatively complex environments, high-quality RSS data is the prerequisite to achieve effective localization. Therefore, a targeted filtering method, two-dimensional double correlation (TDDC) distributed wavelet filtering, is proposed in this paper, aimed at achieving a better filtering effect.

TDDC wavelet filtering is more focused on time-series data correlation than simply wavelet filtering. In order to estimate characteristics of the random disturbances, we refine the detailed coefficient in the wavelet coefficients by analyzing the correlation of the detailed components after wavelet decomposition. At the same time, two kinds of transverse correlation coefficients are calculated to avoid the pseudo-correlation problem [4,5], and further improve RSS data processing.

**Figure 1:**
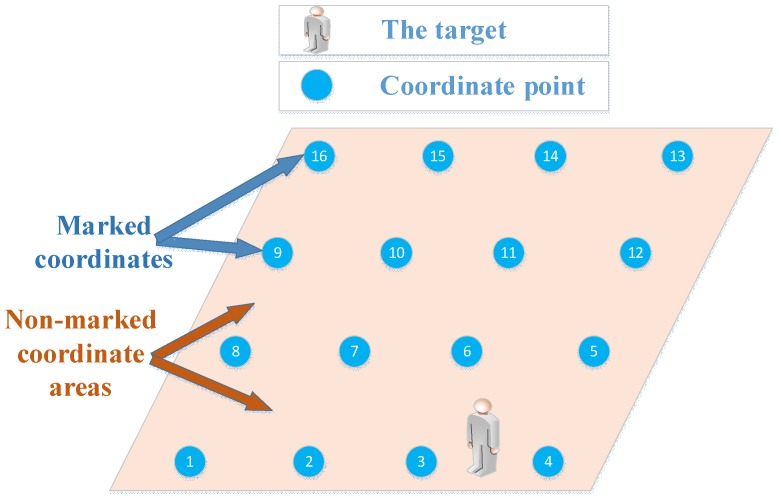
An illustration of target locating at the non-marked coordinate position.

Given the fixed mathematical model for links in a household environment, it is difficult to correctly represent the relationship between the change of the RSS and the change of the target location. Therefore, it is a good choice to adopt a localization method based on machine learning. However, sometimes the links are not sensitive to target locations or are even “blind links”, which will affect model training. Additionally, the number of the collected training samples is very limited, which makes it difficult to cover all the possible sample patterns. In the actual monitoring process, the target position is arbitrary; it will not always locate at the marked coordinates. When the target locates between the marked coordinates (non-marked coordinate areas) the model must have the ability to output the results closest to the actual target location. One example is shown in Figure 1. 

When a target stands at a position between coordinates 3 and 4, a model with poor generalization ability may locate it at coordinate 10 or some other coordinates, which are far from the actual position. But we expect the model to output coordinate 3 or 4, which is nearest to the actual position of the target. In this regard, the Adaboost.M2 [6,7] ensemble learning model based on the Gini decision tree (GDTE) is proposed in this paper.

In order to verify and evaluate the proposed filtering method and localization model, some DFL experiments have been performed in two different ordinary drawing rooms. Since the localization problem is transformed into the multi-classification problem in this manuscript, all the algorithms discussed always return the coordinates of a marked location to represent the target position. Compared with the filtering methods and localization models based on machine learning commonly employed in this field, the proposed TDDC distributed wavelet filtering and the GDTE localization model have some obvious advantages. The main contributions of the paper can be summarized as follows:

(1) With studies of correlations and pseudo-correlations of time-series data, a TDDC distributed wavelet filtering algorithm is proposed, which can better refine the detailed coefficient in the wavelet coefficients, and obtain more accurate characteristics of the interference to achieve the adaptive filtering threshold. It can also preserve the normal fluctuations, as much as possible. Compared with other common filtering methods in this field, the filtering algorithm provides more excellent RSS data for the DFL process and improves the localization accuracy rate.
(2) GDTE can improve the unsatisfactory localization results of single machine learning models, which do not have enough generalization ability and adaptability. The GDTE localization model chooses the more helpful sample attributes for localization and pay more attention to the samples that are more difficult to classify. These characteristics enable the model to deal with the complex relationship between the RSS and the target location. At the same time, weights are updated for RSS samples and weak classifiers (decision trees in this paper). Then, multiple classification results with different weights are generated and the final result is judged by voting. This mechanism can make the model have stronger generalization ability.
(3) We conducted experiments with the hardware test bed in different drawing room environments and evaluated the proposed schemes extensively.

The rest of the paper is structured as follows: Section 2 reviews the related work on device-free wireless localization and RSS filter. Section 3 introduces the system architecture and the motivation behind the proposed schemes. Section 4 presents the detailed methodology of the two-dimensional double correlation distributed wavelet filtering algorithm and the Adaboost.M2 ensemble learning localization model based on the Gini decision tree. Section 5 validates the proposed schemes with extensive experimental evaluations. Finally, the conclusions are drawn in Section 6.

## 2. Related Work

In view of the great significance and broad application prospects in the field of security monitoring and special personnel monitoring, the DFL based on RF network is becoming a research hotspot. Youssef [8] and Zhang [9,10] were the first to propose the concept of device-free localization which establishes the foundation for subsequent research.

Currently, some researches are devoted to establishing a mathematical model that can represent the relationship between the target location and the link RSS value. Wilson and Patwari proposed the RTI approach based on the differential RSS measurements [11,12,13] via the reconstruction of the tomography image for the locations of target. Zhao et al. proposed an RTI model based on the least square variance [14]. Banerjee et al. [15] employed variance-based RTI in target tracking and localization. Kaltiokallio et al. established a fade level-based spatial model for radio tomographic imaging [16]. Lei et al. proposed a new elliptical model for DFL [17], and Yuan et al. improved it [18]. Zhang et al. [19,20] proposed geometrical methods based on the influential links. Xiao et al. proposed a nonlinear optimization model and an outlier rejection method based on the geometrical positional relationship among links [21]. The models based on the exponential-Rayleigh were proposed and used by Guo et al. [22,23,24,25]. These researches represented the relationship among the change in the target position, the distance between the target and the link, and the RSS value from different aspects. Similarly, Hong, Wan and some other researchers have also put forward corresponding localization models and feature extraction methods [26,27]. In addition, methods based on statistical learning also require model-based algorithms. Song et al. [28] proposed a Gaussian process model enabled particle filter for DFL. Savazzi et al. [29] studied the diffraction principle to deal with the average path loss, and proposed a modified stochastic Bayesian approach for real-time target localization. Wang et al. proposed a saddle model [30] and improved ellipse model [31], and achieved localization through improved Bayesian filtering and particle filtering. Ruan et al. presented and realized a data-driven approach which is based on the Gaussian Mixture Model (GMM)-Hidden Markov Model (HMM) and k Nearest Neighbor (KNN)-HMM model [32].

When the environment becomes relatively complex, the fixed model-based DFL methods often fail to achieve a good localization effect. Therefore, some scholars have also proposed localization methods based on machine learning. Initially, Youssef started the DFL research with a method similar to fingerprint identification [33]. Aly et al. [34] leveraged an automation tool for fingerprint construction to study modified scenarios for WiFi-based DFL. Chiang et al. [35] proposed a modified fuzzy SVM for DFL. Wagner et al. employed the various artificial neural network models to achieve the DFL process [36]. Wang et al. proposed a DFL method based on deep learning [3]. Zhang et al. implemented the DFL via an extreme learning machine with parameterized geometrical feature extraction [37]. Ke et al. have also carried out the similar research recently [38,39]. However, the generalization of a single machine learning models is not strong enough, which makes them poorly adaptable to the environment. Additionally, sometimes, unwanted links also affect the training process and localization results.

In pre-processing process, the most important thing is to filter the RSS data properly, but this process is rarely explored in depth. Most of the related literature pays more attention to research on the localization model, and adopts some simple or untargeted filter, such as moving average, Gaussian filtering, common wavelet filtering and so forth [2,3]. It is difficult to obtain suitable filter templates or cut-off frequencies for these filters in different environments. Some adaptive threshold filters, such as some wavelet filters, do not take into account the correlation between normal fluctuations and random disturbances mixed into the RSS data. Therefore, it is difficult for them to effectively filter random disturbances and preserve the valid RSS data as much as possible.

Based on the above discussion, a two-dimensional double correlation (TDDC) distributed wavelet filtering and the localization model of Adaboost.M2 ensemble learning model based on the Gini decision tree (GDTE) are proposed in this paper. The TDDC filtering method is chosen in view of the fact that the occurrences and amplitudes of the disturbances are random, and it is difficult to estimate their characteristics. Compared with a single machine learning model, the GDTE localization model has stronger generalization ability and higher positioning accuracy.

## 3. System Architecture and Motivation

The overall architecture of the DFL algorithm based on the two-dimensional double correlation distributed wavelet filtering (TDDC) and the Adaboost.M2 ensemble learning model based on the Gini decision tree localization model (GDTE) is introduced in this part. The main idea and the calculation process of the TDDC are also described in this part.

### 3.1. System Architecture

**Figure 2:**
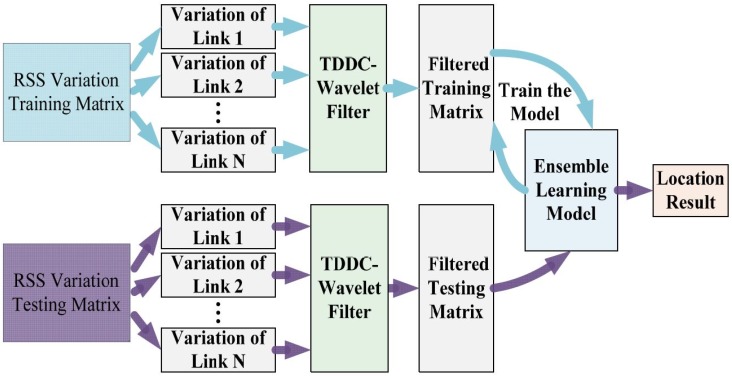
The overall architecture of the DFL algorithm proposed in this paper.

The overall architecture of the DFL algorithm proposed in this paper is shown in Figure 2. The localization process includes online and offline stages. In the offline stages, we collect the RSS data for the training of the TDDC and GDTE models. In the online stage, the trained localization models begin to work when target is at any position in the monitored area. But before that, we need to do some preparations. We arrange a certain number of RF sensors around the monitoring area according to its size, and mark coordinates at certain intervals in the area where the target may appear. Then we collect RSS data of all RF links when the personal target is at different coordinates and organize them into an RSS training sample matrix.

In the offline phase, the TDDC wavelet filtering method is applied to each RSS training sample to filter the random disturbances and reserve the helpful RSS fluctuations. Then we train the GDTE localization model using the filtered RSS samples to update the model parameters. In the online stage, the collected RSS test samples are also filtered by the TDDC distributed wavelet filter, and then the filtered test samples are fed to the trained GDTE model to get location information. 

### 3.2. Main Idea of TDDC Distributed Wavelet Filtering

The key of the TDDC algorithm focuses on multiple correlations of the detailed wavelet coefficients rather than the wavelet transform itself. 

Wavelet analysis can not only express the time domain characteristics of the data, but also express the frequency domain characteristics. The wavelet transform represents a signal as the approximation coefficients (ca) and the detailed coefficients (cd) on different wavelet scales. However, not all of the information provided by the cd is interference; some information helpful for localization may also be included. Therefore, we need to refine the cd in order to obtain the detailed coefficients that can estimate the characteristics of the random disturbance more accurately.

**Figure 3:**
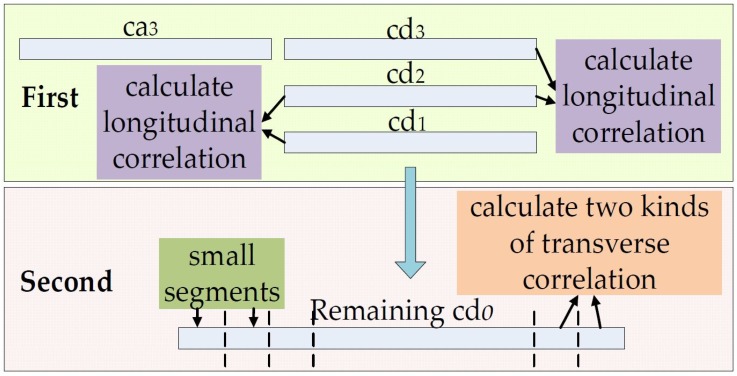
The two key steps of the TDDC wavelet filtering.

The main tasks of TDDC are to obtain the detailed coefficients that can be used to estimate the random disturbance characteristics and get the adaptive filter threshold. Figure 3 expresses the two key steps of the algorithm. 

First, we need to eliminate the obvious target motion components contained in cd*_o_* by calculating the longitudinal correlation coefficients of the cd*_o_* in adjacent wavelet levels, where *o* is the index of wavelet decomposition level. Second, we divide the remaining detailed coefficients into small segments, and calculate the transverse time shift and the transverse zero-time shift correlation coefficients for the adjacent segments. We avoid the pseudo-correlation problem [4,5] by analyzing two kinds of transverse correlation coefficients, and further remove the target motion components. Then the random disturbance characteristics can be estimated better by calculating the final remaining detailed coefficients and an adaptive filter threshold can be achieved. Finally, the filtered RSS data can be recovered through wavelet reconstruction.

The theory of calculating the longitudinal correlation coefficients is as follows: the energy of the available information is relatively concentrated in the wavelet domain, which results in that the absolute values of the wavelet coefficients being relatively large after the wavelet decomposing. However, the energy of the disturbance signal is relatively decentralized, which results in that the absolute values of these wavelet coefficients being relatively small. For a complete theory of the longitudinal correlation, refer to Reference [40]. Therefore, the longitudinal correlation coefficient can be used as a threshold to determine which detailed wavelet coefficients are disturbance components and reserve them for the disturbance characteristics estimation.

The remaining detailed coefficients may still contain the information of the target movement. If all of them are used to estimate the characteristics of disturbances and noises, a portion of the available information, which is helpful for the localization, will be lost and the localization accuracy will be affected. Therefore, we propose a method to further process the reserved detailed coefficients by calculating the transverse zero time-shift correlation coefficients. We divide the remaining detailed coefficients into small segments, refer to Figure 3, and ensure that the coefficient number of each segment is small enough. Due to the limited moving speed and continuous trajectory of the target, the correlations of the adjacent segments would be high without disturbance interference. However, if a certain segment contains interference information due to random disturbances, the correlation between this segment and its neighboring segments should be small relatively. This method can be used to filter the detailed coefficients that can better represent the noise and random disturbance.

Because the frequency and amplitude of random disturbances are unknown, the situation that the disturbances cause adjacent small segments to coincidentally have strong correlations should be considered. This is the pseudo-correlation problem [4,5]. If the pseudo-correlation is not taken into consideration, we will miss some random disturbance and noise information. For this reason, transverse time-shifted correlation coefficients of the adjacent segments are proposed in this paper. The detailed implementation process of the algorithm is described in Section 4.

Through the above preprocessing of RSS data, random disturbance characteristics can be better estimated. The precision of the filtering threshold can be improved and the better filtering effects can be achieved.

## 4. Two-Dimensional Double Correlation Distributed Wavelet Filtering and Adaboost.M2 Ensemble Learning Model Based on the Gini Decision Tree

The detailed calculation processes of the TDDC and GDTE are described in this part. And the in-depth explanations are also included.

### 4.1. TDDC Distributed Wavelet Filtering

**Figure 4:**
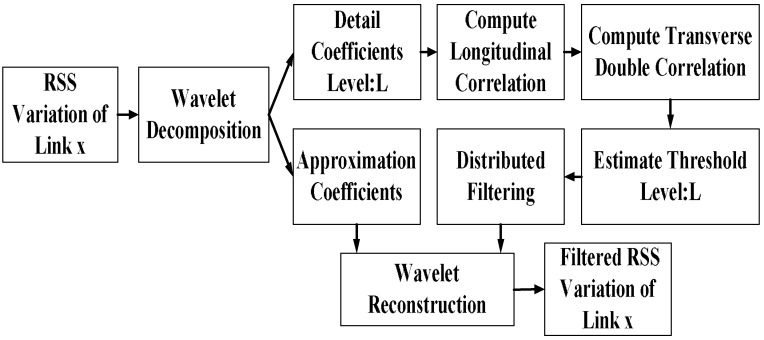
The overall architecture of the TDDC distributed wavelet filtering.

The overall architecture of the filtering algorithm is shown in Figure 4.

The RSS is a 3D mathematical entity that may be expressed as *rss_ijl_* where *i* is the coordinate index (index 0 refers to the background case with no people inside the area); *j* is the index of RSS value and *l* is the link index. In the sample collection process, the RSS value of link *l* is *rss_jl_* at a certain moment, and *rss_j+_*_1*l*_ is the RSS value of the same link at the next moment, and so on. Therefore, the set of training samples collected at coordinate *i* are denoted **R_i_** = {**R_i1_**, **R_i2_**, …, **R_iM_**} = {[*rss_i_*_11_, …, *rss_i_*_1*l*_], [*rss_i_*_21_, …, *rss_i_*_2*l*_], …, [*rss_iM_*_1_, …, *rss_iMl_*]} where M is the total number of the collected samples at coordinate *i*, and the corresponding labels of the set are **Y_i_** = {*y*_1_, *y*_2_, …, *y_M_*} where *y*_1_ = *y*_2_ = … = *y_M_* = *i*. Then all training sample sets collected at every coordinate are denoted by **R** = {**R_1_**, **R_2_**, …, **R_N_**} where N is the number of marked coordinates, and their corresponding labels are **Y** = {**Y_1_**, **Y_2_**, …, **Y_N_**}. The mean values of the RSS for each link when there is no target in the monitor area are denoted by **R_0_**. The differential training samples are **∆R** = {**R_11_** − **R_0_**, **R_12_** − **R_0_**, …, **R_NM_** − **R_0_**}, which form the training sample matrix. One column of the matrix represents a training sample. We process the test samples with the same method.

First, we use wavelet decomposition to process the RSS data with a decomposition level of *o*. For the results in this paper, we take *o* = 4. The wavelet mother function we utilize is the Daubechies wavelet and we adopt the wavelet basis Db1. After the decomposition, we obtain the approximation coefficient vector **ca_4_** and the detailed coefficient vectors **cd_1_**, **cd_2_**, **cd_3_**, and **cd_4_**. 

Next, we calculate the correlation coefficients **corr_o_** for the detailed coefficients of adjacent wavelet levels. The **corr_o_** is obtained by element-wise product of the **cd_o_** and **cd_o+1_** and the product symbol is represented by ⊙:

(1) $${\mathrm{corr}}_{o}=cd_{o}⊙{\mathrm{cd}}_{o+1}$$

where *o* is the index of the wavelet decomposition level. Then, we calculate the energy of the detailed coefficients, *Pcd_o_*, and the energy of the correlation coefficients, *Pcorr_o_*, via:

(2) $$Pcd_{o}=\sum_{g=1}^{n}{\mathrm{cd}}_{o}{}^{2}(g)$$

(3) $$Pcorr_{o}=\sum_{g=1}^{n}{\mathrm{corr}}_{o}{}^{2}(g)$$

where *n* is the number of detailed coefficients for each level and *g* is the index. Finally, we obtain the normalized longitudinal correlation coefficients, **corrn_o_**, based on the two energy parameters:

(4) $${\mathrm{corrn}}_{o}={\mathrm{corr}}_{o}\cdot \sqrt{\frac{Pcd_{o}}{Pcorr_{o}}}$$

The purpose of this step is to ensure the **corr_o_** have the same energy level as the detailed coefficients, which allows them to be compared.

Then we get the remaining detailed coefficients, **cds_o_**, via:

(5) $$\left\{ \begin{array}{l} {\mathrm{cds}}_{o}(g)=0,\;\mathrm{if}\;\left|{\mathrm{cd}}_{o}(g)\right|\geq \left|{\mathrm{corrn}}_{o}(g)\right|\\ {\mathrm{cds}}_{o}(g)={\mathrm{cd}}_{o}(g),\;\mathrm{if}\;\left|{\mathrm{cd}}_{o}(g)\right|<\left|{\mathrm{corrn}}_{o}(g)\right| \end{array}\right.$$

Most coefficients of the **cds_o_** can be regarded as disturbances and noises related.

**Figure 5:**
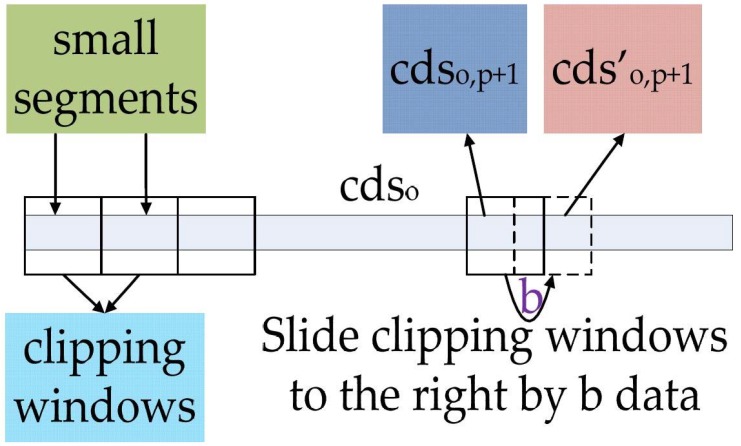
The process schematic diagram of sliding clipping window.

In order to obtain the appropriate filtering threshold, we will refine the **cds_o_** in the following steps. Divide the **cds_o_** into *U* equal segments, referred to as the “small segments” in Figure 5, and assume each small segment contains *d* coefficients, where *U* ≥ 2 and *d* = 10 in this paper. The transverse zero time-shift correlation coefficients *R1_o_*_,*p*_ is obtained using the **cds_o_** of segment *p* and its adjacent segment *p* + 1:

(6) $$R1_{o,p}=\frac{\mathrm{Cov}({\mathrm{cds}}_{o,p},{\mathrm{cds}}_{o,p+1})}{\sqrt[{}]{\mathrm{Var}({\mathrm{cds}}_{o,p})\times \mathrm{Var}({\mathrm{cds}}_{o,p+1})}}$$

where Cov and Var are the covariance and variance of the corresponding **cds_o,p_** and *p* is the index of the small segment. 

Assume that the monitor area is noiseless; all the **cds_o_** coefficients are then only related to the movement of the target. If the speed of the target movement is limited and the number of coefficients in each small segment is very small, then the correlation of the adjacent segments will be strong. This means that the value of *R1_o_*_,*p*_ is relatively larger. However, once random disturbances occur, they will weaken the correlations, which means that the value of *R1_o_*_,*p*_ will be relatively small.

In order to verify the correlations between two small segments more accurately, the pseudo-correlation problem [4,5] must be avoided. We slide the clipping windows of the small segments in time sequence, as shown in Figure 5. 

In the proposed method, the clipping windows of **cds_o,p+1_** are slid to the right by *b* data points, while the sizes of the clipping windows are kept unchanged. The obtained new small segments **cds′_o,p+1_** will be used as parameters for calculating the transverse time-shifted correlation coefficient *R2_o_*_,*p*_ via:

(7) $$R2_{o,p}=\frac{\mathrm{Cov}({\mathrm{cds}}_{o,p},{\mathrm{cds}}_{o,p+1}^{\prime })}{\sqrt[{}]{\mathrm{Var}({\mathrm{cds}}_{o,p})\times \mathrm{Var}({\mathrm{cds}}_{o,p+1}^{\prime })}}$$

Note, *b* is less than or equal to *d*/2, and *d* is the length of small segment. Through the above processing, the coefficients in **cds_o,p+1_** and **cds’_o,p+1_** related to the random disturbances and the helpful fluctuations will both change. Thus, if the *R1_o_*_,*p*_ is pseudo-correlational, *R2_o_*_,*p*_ will change compared to *R1_o_*_,*p*_. If *R2_o_*_,*p*_ changes little or does not change, the *R1_o_*_,*p*_ obtained before can be regarded as the pseudo-correlation, which means that the coefficients in **cds_o,p_** contain disturbance components. Of course, if *R1_o_*_,*p*_ itself is very small, it can be directly demonstrated that the coefficients in **cds_o,p_** contain disturbance components.

Through the above-mentioned double correlation processing, the negative effect of pseudo-correlation can be minimized and the detailed wavelet coefficients related to the disturbances can be chosen more precisely. 

The next step is to refine the detailed coefficients according to *R1_o_*_,*p*_ and *R2_o_*_,*p*_, in order to estimate the disturbance characteristics. We select the segments causing the smallest *R1_o_*_,*p*_ and |*R1_o_*_,*p*_ − *R2_o_*_,*p*_| to form the new segments, which are formally shown in Equations (8)–(11):

(8) $$Rc_{o,p}=\left|R1_{o,p}-R2_{o,p}\right|$$

(9) $${\mathrm{cdrc}}_{o}={\mathrm{cds}}_{o,p},\;\mathrm{where}\;Rc_{cd}{}_{{}_{o,p}}=min(Rc_{o,p})$$

(10) $${\mathrm{cdr}}_{o}={\mathrm{cds}}_{o,p},\;\mathrm{where}\;R1_{cd_{o,p}}=min(R1_{o,p})$$

(11) $${\mathrm{cdc}}_{o}=\left\{{\mathrm{cdrc}}_{o},{\mathrm{cdr}}_{o}\right\}$$

where the **cdc_o_** is the new detailed wavelet coefficient array. 

According to improvements of the empirical formula, we can estimate the random disturbance characteristics and the filtering threshold according to:

(12) $$\sigma_{o}=2\times \mathrm{median}(1/m\sum_{i=1}^{m}{\mathrm{cdc}}_{o})/0.6745$$

(13) $$thr_{o}{=\sigma }_{o}\sqrt{2\mathrm{lg}(n)}$$

where m is the number of data points in segment **cdc_o_**.

The frequency of the coefficients in **cd_1_** is too high to be caused by the target, so these coefficients can all be set to zero. The detailed coefficients of other levels can be filtered through the above filtering threshold. The distributed filtering process is then:

(14) $$\left\{ \begin{array}{l} {\mathrm{cdf}}_{1}=0\\ {\mathrm{cdf}}_{o}=\left\{ \begin{array}{l} {\mathrm{cd}}_{o}(g)=0,\;\mathrm{if}\;\left|{\mathrm{cd}}_{o}(g)\right|\geq {thr}_{o}\\ {\mathrm{cd}}_{o}(g)=\left|{\mathrm{cd}}_{o}(g)\right|-thr_{o},\;\mathrm{if}\;\left|{\mathrm{cd}}_{o}(g)\right|\geq thr_{o} \end{array}\right. \end{array}\right.$$

where **cdf_1_** is the detailed wavelet coefficient vector of the first level, filtered to zero, and **cdf_o_** are the filtered detailed wavelet coefficient vectors of other levels. 

Finally, the low-frequency coefficients and the filtered detailed coefficients are combined into the new wavelet coefficients to recover the filtered RSS data through wavelet reconstruction. All the RSS training samples and testing samples should be filtered by the above method.

### 4.2. GDTE Localization Model

**Figure 6:**
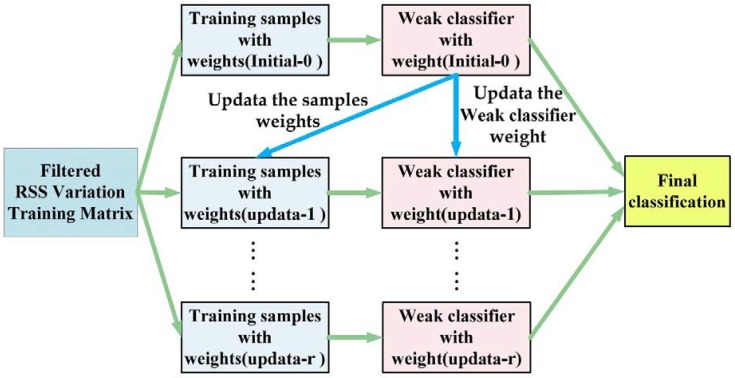
The schematic diagram of the ensemble learning model.

The ensemble learning model is a strong classifier composed of multiple weak classifiers, as Figure 6 shows. It is r round during the iteration process in the figure. We update the weights of the samples and the weak classifiers according to the classification error rate of each iteration. Then, we make the next weak classifier focus on the samples which have been misclassified in the last iteration by increasing their weights. In the testing phase, the final classification results of test samples are determined by comprehensive statistics on the all weighted weak classifiers.

The decision tree is chosen as a weak classifier in this paper. As mentioned above, when the environment is relatively complex, the relationships between the RSS variation of each RF link and the change of the target location are indefinite; the importance of each attribute (each link) of the RSS sample is different. Therefore, in order to generate a suitable decision tree, an appropriate metric needs to be chosen to evaluate importance of the attributes. The Gini coefficient indicates the purity of the sample categories. And the more uniform the sample categories are, the smaller the Gini coefficient is. The attributes that minimize the Gini coefficient are regarded as the optimal branch attributes for decision tree growth. The Gini coefficient is defined by:

(15) $$Gini(A)=\sum_{c=1}^{v}\frac{S_{c}^{}}{E}\left[1-\sum_{b=1}^{N}{\left(\frac{S_{{}_{bc}}^{}}{S_{c}^{}}\right)}^{2}\right]$$

where *V* is the branch number of one internal node (it means that the samples in it can be classified into *V* categories), *E* is the total number of the samples contained in the node, and *N* is the total number of coordinates in the monitored area (the same as the total number of the RSS sample categories). Assume that link *A* of the RSS sample is chosen as a branch attribute for decision tree growth and the samples contained in the node can be classified into *V* categories, then the sample number of each category is *S_c_*, which also acts as a label of this category. *S_bc_* is the number of RSS samples belonging to coordinate *b* in each *S_c_*. When link *A* is considered as a branch attribute, it can be seen that the more uniform (that is, the higher purity) the categories of the RSS samples contained in the internal node of the decision tree are, the smaller the Gini coefficient of the attribute *A* is. With this calculation, we can choose the links that are more sensitive, and thus more helpful to localization, as the best attributes for the current classification. 

After formulating the rules of generating the weak classifiers, we need to build a final ensemble learning model based on the Adaboost.M2 algorithm. Starting from the first decision tree, the new decision tree is generated and trained through iteration. During the initialization phase, the weight of a mislabel *y* of sample *q* can be written as:

(16) $$w_{q,y}^{1}=\frac{1}{M(N-1)}$$

where *q* is the index of the RSS sample, *N* is the number of coordinates, and *M* is the number of training samples collected at each coordinate. *N* − 1 in the denominator is the total number of mislabels.

For each round of iteration, the RSS sample weights are updated, and the corresponding decision tree weights are obtained according to the following equations:

(17) $$\left\{ \begin{array}{l} W_{q}^{t}=\sum_{y\neq y_{q}}^{}w_{q,y}^{t}\\ D_{q}^{t}=\frac{W_{q}^{t}}{\sum_{q}^{MN}W_{q}^{t}}\\ w_{q,y}^{t+1}=w_{q,y}^{t}\times \beta_{t}{}^{\left(1/2)(1+h_{t}({\mathrm{RSS}}_{q},y_{q})-h_{t}({\mathrm{RSS}}_{q},y)\right)} \end{array}\right.$$

(18) $$e_{t}=\frac{1}{2}\sum_{q}^{MN}D_{q}^{t}(1-h_{t}({\mathrm{RSS}}_{q},y_{q})+h_{t}({\mathrm{RSS}}_{q},y))$$

(19) $$\beta_{t}=\frac{e_{t}}{1-e_{t}}$$

(20) $$HW_{t}=\log(\frac{1}{\beta_{t}})$$

where $w_{q}^{t}$ is the sum of the weights of all mislabels of sample *q* in the *t*th iteration, $D_{q}^{t}$ is the weight ratio of sample *q* to all samples in iteration *t*, the mislabel distribution, h_t_(*x_q_*, *y*) is the possibility of the weak classifier getting back a hypothesis *y* for the sample *q* at iteration *t*, where *y* is the mislabel of the *x_q_*, h_t_(*x_q_*, *y_q_*) is the possibility of the weak classifier getting back the correct label *y_q_* for sample *q* at iteration *t*. ε_t_ is the error rate of the *t*th decision tree, which is computed with related distribution $D_{q}^{t}$ over the training examples and incorrect labels obtained in the previous iteration. *HW_t_* is the weight of the hypothesis, such that greater weight is given to a hypothesis with lower error. The final classification result of the ensemble learning model is formulated as:

(21) $$h_{\mathrm{fin}}({\mathrm{RSS}}_{x})={\mathrm{argmax}}_{y\in Y}\sum_{t=1}^{T}(HW_{t})h_{t}({\mathrm{RSS}}_{x},y)$$

That is, for an RSS sample, h_fin_ outputs the label *y* that maximizes the sum of the weights of the weak hypotheses predicting that label. For more detailed description of the Adaboost.M2 algorithm, see [5,6].

Through the above algorithmic processing, an ensemble learning model is generated and trained. During the online phase, the RSS samples are filtered and fed to the localization model one by one. The final localization results can be determined through comprehensive statistics of the classification results returned by different decision trees and their different weights.

## 5. Experimental Evaluation

This section introduces the experimental environments, multiple experimental processes, the result comparing and analyzing with similar algorithms commonly adopted in the DFL field, and demonstrating the advantages of the proposed localization algorithms.

### 5.1. Description of the Experiment

**Figure 7:**
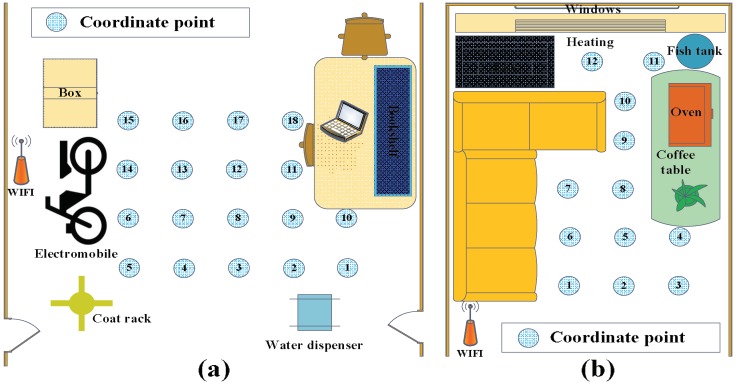
An illustration of the layout of the two experiments. (**a**) The layout of the drawing room 1. (**b**) The layout of the drawing room 2.

The core chip of the RF sensor node is TI’s CC2530 chipset (Texas Instruments corp., Dallas, TX, USA) which could transmit and receive signals in the 2.4 GHz ISM band, and for more information about the node, refer to Reference [41]. According to the size of the experimental monitoring area, some sensor nodes are arranged around the area and communicate with each other through different channels so as to avoid mutual interference. There are 48 RF links in the monitoring area, and the RSS data of all the links are transmitted to a PC through a data collection node, in real time. The layouts of the two experiments are illustrated in Figure 7 and the coordinates are marked every 0.5 m in the area where the target may appear. Eighteen coordinates numbered from 1 to 18 are marked in drawing room 1, and 12 coordinates are numbered and marked from 1 to 12 in drawing room 2. 

In the offline phase, the RSS data of a personnel target standing at each coordinate is collected to form the training sample matrix; in the online phase, the test RSS samples are collected when the target walks from one position to another arbitrarily and stands at each coordinate. Compared to drawing room 1, the noise and disturbances in drawing room 2 are stronger, so the experimental results obtained in drawing room 2 can better show the filtering and localization advantages of the proposed algorithm.

### 5.2. Performance Comparison

**Figure 8:**
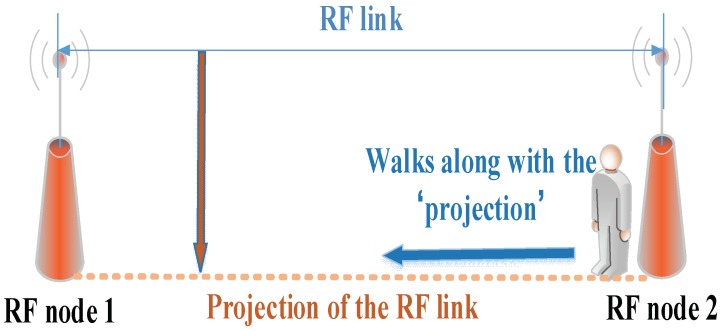
An illustration of the target moving.

First, in the two experiments, we collect the RSS data of a link when the target moves along the projection on the ground of the link from one end to another and observe the changes of the RSS value, as Figure 8 shows.

**Figure 9:**
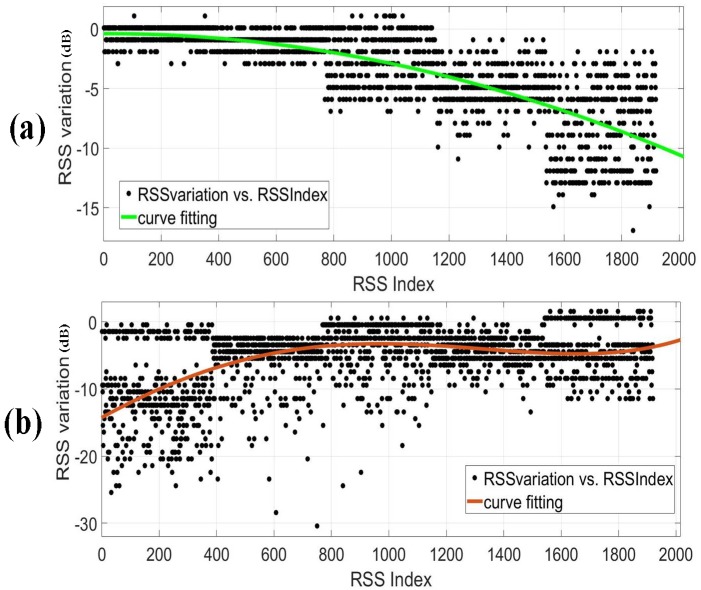
The RSS variation rules of different links. (**a**) The RSS variation of a link in the drawing room 1. (**b**) The RSS variation of a link in the drawing room 2.

Figure 9 shows two instances, the black dots are the RSS values and the color curve is the fitting curve for them. The horizontal axis indicates the sample indexes sorted according to the time of collection and the vertical axis indicates the changes of the RSS values. It can be seen that the RSS variation rules of different links are not uniform for the same target moving trajectory. A fixed mathematical model finds it difficult to represent the relationship between the change of the target location and the RSS variation of the corresponding links, since the relationship varies with different scenarios and links. Therefore, it is a good choice to use machine learning to achieve target position.

Gaussian filtering (Gauss) and moving average filtering (MA), which are commonly adopted in this research field, are compared with the TDDC distributed wavelet filtering. The parameters of the Gaussian filter and moving average filter are adjusted for two different monitoring scenarios to achieve the best results. Other wavelet-based filtering methods are also involved in the comparison. Single correlation soft-threshold wavelet filtering [42,43] (SC), correlation feature scale entropy wavelet filtering [44] (CE), and detailed coefficients zero-setting wavelet filtering [3] (HFC) are compared with TDDC wavelet filtering, respectively. The SC calculates the longitudinal correlation coefficients only, and then obtains the filtering threshold according to the remaining detailed wavelet coefficients. The CE refines the detailed wavelet coefficients by comparing the entropies of **cD_o_**, and then obtains its filtering threshold. The HFC sets all the detailed wavelet coefficients to zero.

Since we cannot measure reliable noiseless RSS data, and the frequencies and amplitudes of the random disturbances and noise are unknown, the traditional signal-to-noise ratio method is not suitable to evaluate the effect of TDDC distributed wavelet filtering. Instead, we feed the filtered RSS data into the same ensemble learning model and evaluate the filtering performance by comparing the final localization accuracy directly. Their performances are compared through calculating the accuracies of the localization results for multiple test samples. 

**Figure 10:**
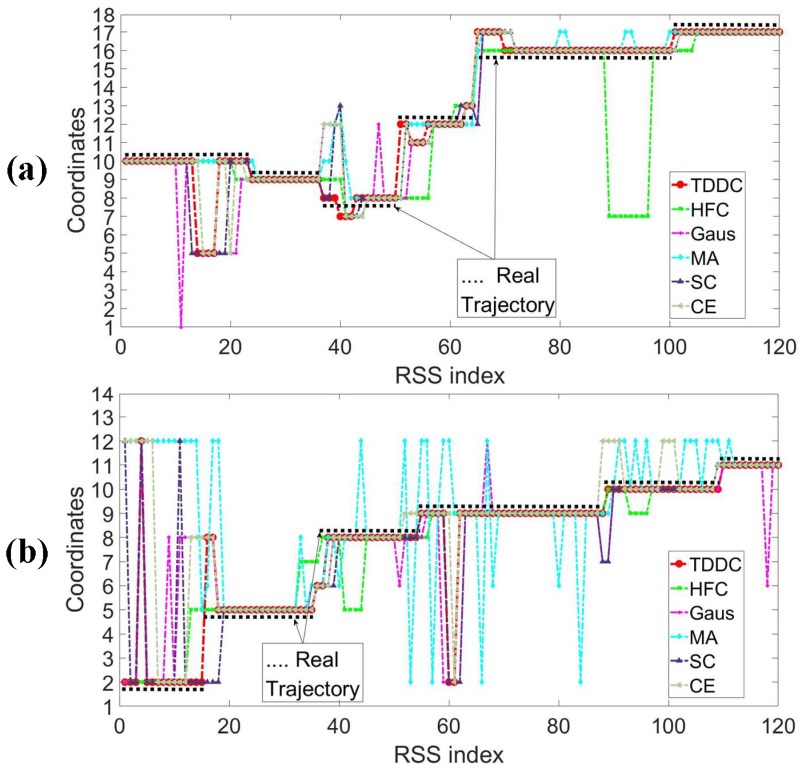
The classification results of the two testing samples filtered by different wavelet filter. (**a**) The testing sample was collected in drawing room 1. (**b**) The testing sample was collected in drawing room 2.

Figure 10 shows classification results of two test samples filtered by different wavelet filters. The two kinds of samples were collected when the target walked from one position to another arbitrarily, which can be regarded as the generic trajectories. But the coordinates of arbitrary positions cannot be recorded precisely, so we use their closest mark coordinates to represent them.

The two samples are recorded as the coordinates 10-9-8-12-16-17 (Figure 10a) where the target walked through in drawing room 1 and the coordinates 2-5-8-9-10-11 (Figure 10b) in drawing room 2. The RSS data collected at non-marked coordinates are also included in the two samples. 

Since this paper transforms the localization issue into a multi-classification issue, the final localization result of each test sample is a coordinate index. If the test samples collected at non-marked coordinates are classified as the coordinate point which is closest to the actual position of the target, the location result will be regarded as correct. We use this rule to calculate and compare the localization accuracy and generalization ability of various algorithms in this paper.

It can be seen from the Figure 10, comparing with the other filtering methods, the located trajectory (solid red dots) using the TDDC has fewer “glitch impulses” and matches the actual moving track of the target (solid black dots) more closely. In terms of filtering RSS data, the TDDC filtering method has more advantages compared with the above filtering methods and can help obtain more accurate location results.

Table 1 records the statistical results for the accuracy rates of multiple test groups. In the same environment and with 0.5 m resolution, the localization accuracy is higher when the RSS data is filtered by TDDC wavelet filtering. 

We have also tested GDTE localization model, and compared the localization effects with the fingerprint model (FP), the SVM model, and the deep neural network (DNN) model which are commonly used in this field. Experiments were carried out with and without TDDC filtering. After repeating the experiments of the deep neural network model, the structure is finally identified as the four neural network layers and the neurons are fully connected. We added a regularization term and a dropout layer to avoid the local minimum and over fitting problems. Table 2 shows the classification accuracy rate of the multi-group test samples without filtering. It can be seen that compared with other models, the GDTE model has a higher accuracy rate in different test environments under the resolution of 0.5 m.

**Figure 11:**
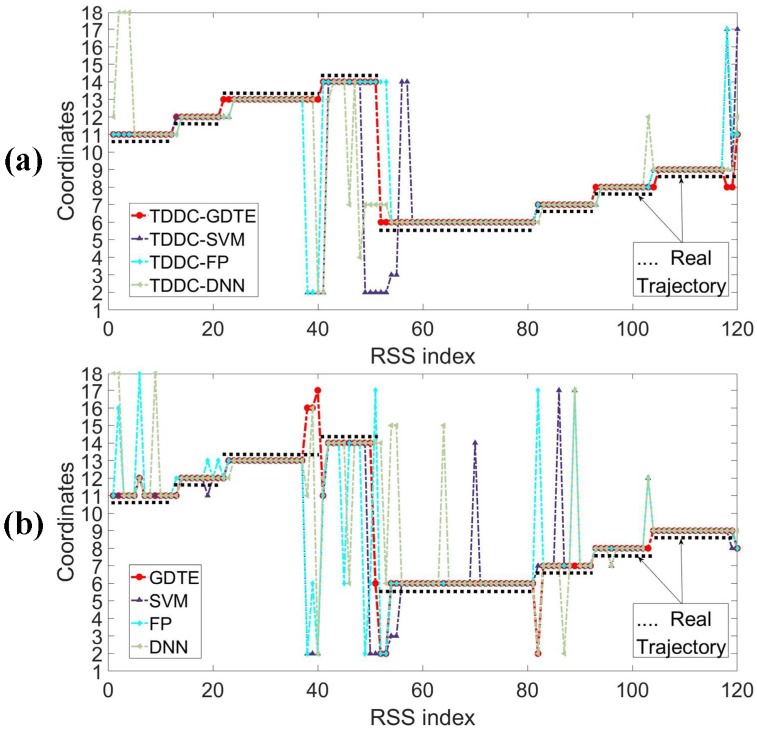
The classification results of a testing sample processed by the different localization models with and without the TDDC wavelet filter. (**a**) The testing sample was filtered by the TDDC wavelet filter. (**b**) The testing sample was not filtered.

The samples recorded as 11-12-13-14-6-7-8-9 in drawing room 1 are classified by different localization models with (Figure 11a) and without (Figure 11b) filtering. The localization results are shown in Figure 11. 

As can be seen from the figure, the results of the GDTE model (solid red dots) are more consistent with the actual moving track of the target (solid black dots), no matter whether RSS data is filtered or not. As the target moves continuously, the classification results of the samples which are collected at non-marked coordinates processed by the GDTE model are more consistent with the expectation of the nearest coordinate compared to other localization models. Table 3 shows the statistical results of the accuracy rate of multi-group test samples processed by the above localization models with the TDDC filtering. From this, we can draw the same conclusions as above. From the comparison between Table 2 and Table 3, it also can be found that the localization accuracy rate of the same localization model has been improved more or less through preprocessing the RSS data with the TDDC wavelet filtering.

**Figure 12:**
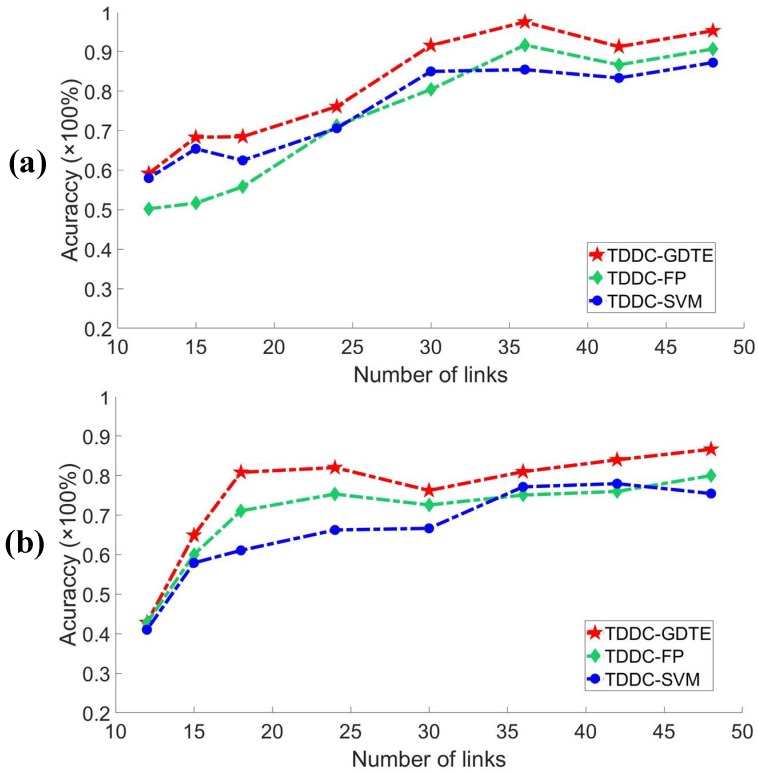
The localization accuracy varying with different numbers of links. (**a**) Experiment in STATE1. (**b**) Experiment in STATE2.

The number of links in the monitoring area usually affects the localization results for the DFL method. We compared the localization accuracy rates of the different localization models with TDDC filtering when the monitor area has a-different-number links. We only considered the situations where the accuracy rates are greater than 60%. Since the DNN structure and parameters need be recalibrated according to the variation dimensions of the samples, only the accuracy rates of the TDDC-GDTE, TDDC-SVM, and TDDC-FP are compared. Figure 12 shows that due to the different multipath effects in different environments, the influence of the number of links on localization are also quite different. 

The more complex the environment is, the more difficult it is to estimate the importance of the links for localization. However, generally the higher the number of links, the higher the accuracy rate is. In the case of different numbers of links, the TDDC-GDTE localization method proposed in this paper has the advantages in both the accuracy rate and stability, especially when the accuracy rate is greater than 70%.

**Figure 13:**
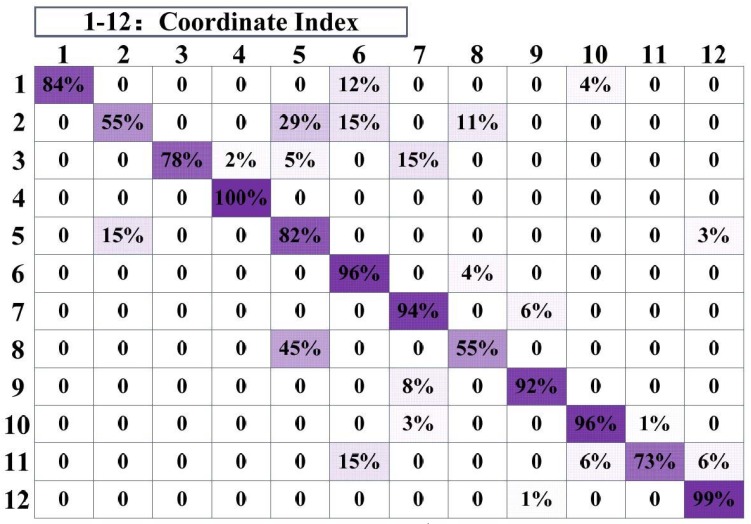
The statistics of the localization accuracy when the target passes through different coordinate points in drawing room 2.

In statistics, we also found that the localization accuracy will be relatively low when the target moves through some specific locations. This phenomenon occurs repeatedly near several specific coordinates. Some of them are close to obstacles, but others are not. We suspect that in a complex environment, due to the serious multipath effect and the noise caused by the target’s movement, the RSS value may change more sharply even over a short distance in those specific areas. This causes the positioning result to be non-ideal. Figure 13 shows the probability of mistakenly locating one coordinate to another. The numbers surrounding the matrix are the coordinate indexes in drawing room 2. The diagonal data of the matrix indicates the probability of correctly locating for each coordinate point, and other data in the same row indicates the probability of mistakenly locating. The sum of the data in each row is equal to 100%. It can be seen that the accuracy is low when the target is near coordinate points 2 and 8. There are obstacles such as furniture and appliances near coordinate 8, but there are no obstacles around coordinate 2.

## 6. Conclusions

This paper explores the two-dimensional double correlation distributed wavelet filtering method and the Adaboost.M2 ensemble learning localization model, based on the Gini decision tree, in order to implement DFL in relatively complex environments. First, we analyzed the relationship between the RSS change of the RF link and the change of the target location in the complex environment. We pointed out that it is difficult to represent this relationship with fixed mathematical models, and focused on the analysis of the RSS data filtering problem, which has great influence over the location results. Compared with the traditional filters and other wavelet-based filtering methods, the TDDC wavelet filtering method not only filters the random disturbances and noise, but also preserves the RSS data that are helpful for localization, as much as possible. The GDTE localization model has a higher localization accuracy and stronger anti-interference ability than the single machine learning models. Experiments have been conducted in two drawing rooms with different furnishings. A localization accuracy rate of 95.28% was obtained in the less disturbing environment. In the environment with stronger disturbances, the TDDC-GDTE was still able to achieve an 87.67% localization accuracy rate. In the case of reducing the number of links in the monitoring area, the performance of the proposed scheme is also better than other algorithms. 

We found that the localization results when the target is near some coordinates in the monitoring area are not always satisfactory. Some of these coordinates are surrounded by obstacles but others are not close to any obstacles. In this regard, we have also made an assumption that the RSS values of some links change sharply in those regions where the multipath effect is serious. Of course, in addition, there are still many problems that need to be solved in the DFL process for complex environments, such as the small sample problem of the DFL process in a large monitoring area and the problem of multi-target localization. We will try to settle these problems in our future work.

## Figures and Tables

**Table 1:** The statistical results of the localization accuracy rates for multiple test samples filtered by different methods.

Filter Accuracy Scenario	TDDC	SC	CE	HFC	Gaus	MA
Drawing room 1	95.28%	91.50%	93.89%	80.50%	89.50%	86.40%
Drawing room 2	87.67%	81.71%	83.23%	67.82%	80.74%	63.17%

**Table 2:** The localization accuracy of different localization models without filtering.

Filter Accuracy Scenario	GDTE	DNN	FP	SVM
Drawing room 1	90.67%	80.80%	80.77%	82.77%
Drawing room 2	82.49%	72.77%	74.55%	75.91%

**Table 3:** The localization accuracy rate of the different localization models with the same TDDC filtering.

Filter Accuracy Scenario	TDDC-GDTE	TDDC-DNN	TDDC-FP	TDDC-SVM
Drawing room 1	95.28%	84.61%	90.66%	87.22%
Drawing room 2	87.67%	77.11%	81.00%	77.46%
